# GPs’ and pharmacists’ views of integrating pharmacists into general practices: a qualitative study

**DOI:** 10.3399/BJGP.2022.0518

**Published:** 2023-05-16

**Authors:** Ameerah S Hasan Ibrahim, Heather E Barry, Carmel M Hughes

**Affiliations:** Faculty of Pharmacy, Al-Zaytoonah University of Jordan, Amman, Jordan, and researcher, Primary Care Research Group, School of Pharmacy, Queen’s University Belfast, Belfast, UK.; School of Pharmacy, Queen’s University Belfast, Belfast, UK.; School of Pharmacy, Queen’s University Belfast, Belfast, UK.

**Keywords:** general practice, general practitioners, pharmacists, primary health care, qualitative interviews

## Abstract

**Background:**

Practice-based pharmacists (PBPs) have been introduced into general practice across the UK to relieve some of the pressures within primary care. However, there is little existing UK literature that has explored healthcare professionals’ (HCPs’) views about PBP integration and how this role has evolved.

**Aim:**

To explore the views and experiences of GPs, PBPs, and community pharmacists (CPs) about PBPs’ integration into general practice and their impact on primary healthcare delivery.

**Design and setting:**

A qualitative interview study in primary care in Northern Ireland.

**Method:**

Purposive and snowball sampling were used to recruit triads (a GP, a PBP, and a CP) from across five administrative healthcare areas in Northern Ireland. Sampling of practices to recruit GPs and PBPs commenced in August 2020. These HCPs identified the CPs who had most contact with the general practices in which the recruited GPs and PBPs were working. Semi-structured interviews were recorded, transcribed verbatim, and analysed using thematic analysis.

**Results:**

Eleven triads were recruited from across the five administrative areas. Four main themes in relation to PBPs’ integration into general practices were revealed: evolution of the role; PBP attributes; collaboration and communication; and impact on care. Areas for development were identified such as patient awareness of the PBP role. Many saw PBPs as a ‘central hub–middleman’ between general practice and community pharmacies.

**Conclusion:**

Participants reported that PBPs had integrated well and perceived a positive impact on primary healthcare delivery. Further work is needed to increase patient awareness of the PBP role.

## INTRODUCTION

Health workforce shortages and the increasing complexity of caring for older people with chronic conditions have increased pressure on primary care services.^1^^–^^3^ Pharmacists were introduced into general practices to alleviate some of the pressures within primary care (known as practice-based pharmacists; PBPs) by delivering a range of activities.^1^^,^^3^^–^^9^ Five-year PBP pilot schemes have been launched in both England and Northern Ireland (NI) to integrate PBPs into general practice.^7^^,^^10^

Most emerging literature has focused on determining barriers and facilitators to the PBP role in general practice; however, there was little detail in relation to the context of the PBP role.^11^ Additional research was recommended to assess healthcare professionals’ (HCPs) experiences of this new role in general practice and the impact on GP workload.^11^ Exploring such views on PBPs will enhance the understanding of their impact in primary care and help inform the development of the role.^12^ Furthermore, the effectiveness of the primary healthcare team is limited by what HCPs know about each other and each other’s roles.^13^^–^^15^

Little is known about the views of key HCPs, particularly GPs and community pharmacists (CPs), regarding the introduction and integration of the PBP role. A previous cross-sectional study conducted by the authors exploring NI GPs’ views on PBPs highlighted some issues in relation to integration and development of the PBP role that merit further examination.^4^ Therefore, this research aimed to contribute to a comprehensive exploration of GPs’, PBPs’, and CPs’ experiences of working with PBPs, and their views on the integration of PBPs into general practice and their impact on primary healthcare delivery in NI.

## METHOD

A qualitative approach to data collection was adopted using semi-structured individual interviews with GPs, PBPs, and CPs across NI. The study was reported according to the Consolidated Criteria for Reporting Qualitative Research (COREQ) checklist.^16^

### Study population and setting

The perspectives of three key HCPs (GPs, PBPs, and CPs) practising throughout NI were sought (Box 1). As a result of the COVID- 19 pandemic, interviews were conducted individually, via telephone or a virtual meeting platform such as Microsoft Teams.

**Box 1.** Inclusion criteria for the three key healthcare professionals — GPs, practice-based pharmacists, and community pharmacists

**Criteria** GPs working with PBPs in their general practices. PBPs employed (full time or part time) in one of 17 GP federations in NI (see Supplementary Information S5 for further information about the federations and links with PBPs). CPs who had most contact with general practices in which the recruited GPs and PBPs worked.

*CP = community pharmacist. NI = Northern Ireland. PBP = practice-based pharmacist.*

### Sampling and recruitment

Purposive and snowball sampling were used to obtain GPs’, PBPs’, and CPs’ perspectives, and to enhance recruitment. Previous studies have found that, if a general practice agrees to participate in a study, associated community pharmacies will also agree.^17^^,^^18^ Therefore, sampling was initiated by contacting three GPs (known to two of the authors), to ask them to identify potential GPs and PBPs whom they knew and who might be interested in the study. These GPs asked permission from these potential participants to share their contact details with the researcher (the first author). In turn, during the interviews, a recruited GP and PBP were asked to nominate and suggest the name of a potential CP participant with whom the practice had most contact. The researcher contacted that pharmacist using publicly available contact details. Therefore, triads were recruited, each consisting of a GP, PBP, and CP, from across the five Health and Social Care Trust areas in NI (that is, administrative healthcare areas in NI) with a view to recruiting up to three triads per area and one triad per practice.

**Table:** How this fits in

Little is known about primary healthcare professionals’ views on the impact of practice-based pharmacists (PBPs) in general practice. Participants interviewed in this study reported that PBPs had integrated well and perceived a positive impact on primary healthcare delivery. The findings indicated that continued integration would need PBPs, all members of the practice team, and community pharmacists (CPs) to understand each other’s roles well and to communicate clearly to ensure the delivery of efficient PBP-led patient care. A number of areas for development were identified, such as patient awareness of the role and communication pathways between PBPs and CPs.

The researcher invited potential HCP participants by telephone/email. If interested, the researcher provided a formal invitation letter and information sheet. Interviews were conducted with the GP and PBP within the potential triad when both the GP and PBP agreed to participate. If a CP within a triad did not agree to participate, the GP and PBP interviews were retained for analysis and it was noted that a CP could not be recruited.

### Data collection

Interviews were conducted by the researcher between August 2020 and October 2021. Participants provided written informed consent and were offered £50 for participation. Separate topic guides were developed for each HCP group (Supplementary Information S1–S3), based on published literature within the field, and piloted.^5^^,^^12^^,^^19^^–^^29^ All interviews were audiorecorded with the permission of the participant.

### Data management and analysis

All interviews were recorded, transcribed verbatim, and checked for accuracy. To ensure anonymity and confidentiality, each participant was assigned a two-digit identification number reflecting the triad with which the participant was associated (for example Triad 1: GP01, PBP01, CP01). Transcripts were imported and managed in NVivo 12 Pro.

Data analysis, using thematic analysis,^30^ was performed in parallel with data collection by two independent researchers. Data from interviews with each type of HCP were analysed separately to monitor data saturation within each HCP category. Themes were reviewed and refined by the research team. The quality and rigour of research reporting was closely monitored (Supplementary Table S1).

## RESULTS

Eleven triads were recruited from across the five administrative areas (Table 1), at which point data saturation was deemed to have occurred. A CP in one triad from the Western Trust was unable to participate because of COVID-19 vaccination workload; however, the GP and PBP interviews were retained for analysis. Of the 32 interviews conducted, most were undertaken by telephone (*n* = 22), with the remainder using Microsoft Teams.

**Table 1.** A summary of the demographic profile of participating GPs, practice-based pharmacists, and community pharmacists

**Characteristic**	**Value, n (%)^a^**
**GPs (*n* = 11)**	
**Sex**	
Female	5 (45)
Male	6 (55)
**Location of general practice**	
Rural	3 (27)
Suburban	3 (27)
Urban	5 (46)
**Trust area of NI in which general practice was located**	
Belfast	3 (27)
Northern	1 (9)
South-Eastern	2 (18)
Southern	2 (18)
Western	3 (27)
**Years since the GP participant had obtained CCT or equivalent (qualified as a GP), mean (SD)^b^**	16.9 (8.4)

**PBPs (*n* = 11)**	
**Sex**	
Female	8 (73)
Male	3 (27)
**Length of time working within the current general practice**	
<1 year	3 (27)
1–2 years	4 (36)
>2 years	4 (36)
**Number of sessions per week working within the current general practice, mean (SD)**	6.3 (2.3)
**Working arrangements of PBPs in the current general practice (full time, part time)**	
Full time (10 sessions per week)	3 (27)
Part time (<10 sessions per week)	8 (73)
Part time 6–9 sessions	4 (36)
Part time 2–5 sessions	4 (36)
Part time <2 sessions	0 (0)

**CPs (*n* = 10)**	
**Sex**	
Female	3 (30)
Male	7 (70)
**Location of the community pharmacy**	
Rural	2 (20)
Suburban	1 (10)
Urban	7 (70)
**Employment status**	
Employee pharmacist	9 (90)
Employer pharmacist	1 (10)
Self-employed locum	0 (0)
**Number of years practising as a CP, mean (SD)**	18.1 (11.1)

a *Data are n (%) unless otherwise specified.*

b *One of the GPs was a GPST3 registrar. CCT = certificate of completion of training. CP = community pharmacist. HCP = healthcare professional. NI = Northern Ireland. PBP = practice-based pharmacist. SD = standard deviation.*

### Demographic data

In total, 11 GPs, 11 PBPs, and 10 CPs participated (Table 1). The participants’ demographics demonstrated variety in terms of key characteristics relevant to the research as they were recruited from a range of geographical areas (that is, five administrative areas; see Supplementary Information S4 for more details about demographic data). At the time of the study, most GPs (*n* = 8, 73%) had two PBPs working part time in their practices. More than half of participating PBPs (*n* = 6, 55%) had been working as a PBP for >2 years. CPs had been in community practice for an average of 18.1 (standard deviation 11.1) years.

### Main themes from thematic analysis

**Figure 1.**
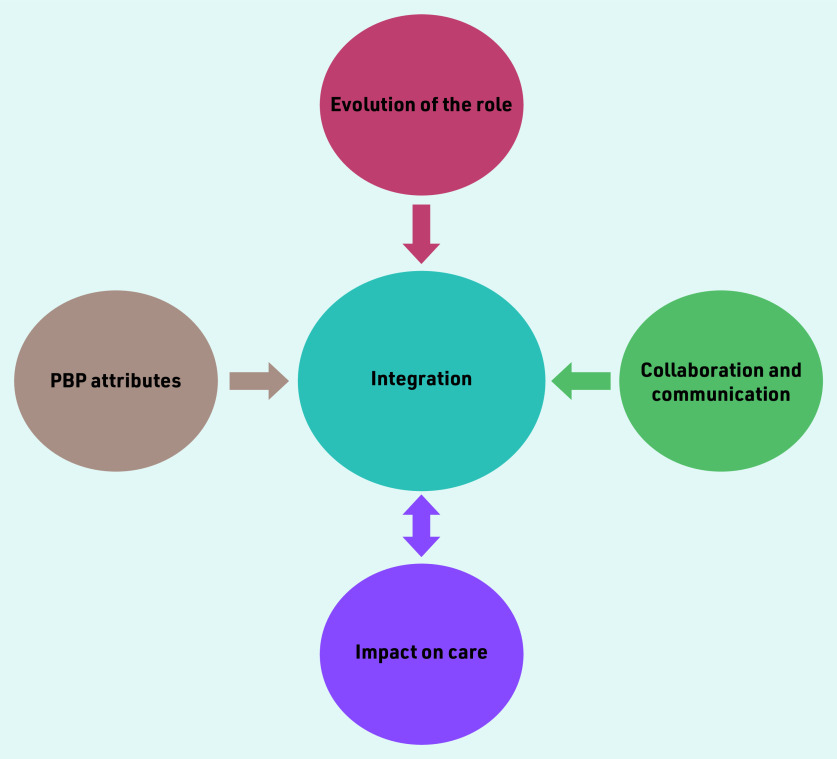
*Four main themes that emerged from thematic analysis of interview data. The double- headed arrow indicates that integration led to an impact on care, and impact on care contributed to better integration. PBP = practice-based pharmacist.*

Thematic analysis revealed four main themes in relation to PBPs’ integration into general practices. This study found that the evolution of the role; PBP attributes; collaboration and communication; and impact on care contributed to integration of PBPs into general practice. Furthermore, integration and impact on care were interrelated (as indicated by the double-headed arrow in Figure 1) in that integration led to an impact on care, and impact on care contributed to better integration.

### Theme 1: evolution of the role

All GPs and PBPs described the wide range of activities undertaken by PBPs that could also differ between practices. They emphasised that the PBP role largely consisted of two main activities: medicines reconciliation of hospital discharge letters and outpatient letters, and medication reviews (see Supplementary Information S5 for definition). The role evolved with time and PBPs began to engage with other tasks, such as independent prescribing and conducting chronic disease review clinics:

*‘They have a wide range of roles … they do the medicine reconciliation from the discharge letters, outpatient letters … do medication reviews … so that would be kind of core work in addition to that they do some of the repeat dispensing prescriptions and also some appropriate acute requests … they would do hypertension clinic, diabetes clinics … so it’s a very busy role and I think it’s a very expanding role as well.’*

(GP11)

PBPs and GPs reported that conducting chronic disease review clinics (see Supplementary Information S5 for definition) in general practice would continue to be an expanding and evolving role for PBPs, with better integration as they undertook more clinical roles. However, there were workload pressures and time constraints that sometimes prevented this happening, leading to prioritisation of medication reconciliation and medication reviews:

*‘I feel practice pharmacists have a very good knowledge base and very good capability and skills to be able to contribute to the long-term management review clinics but sometimes that is not possible because their time is needed elsewhere in the medication reconciliation process and medication reviews in general.’*

(PBP05)

PBPs reported that time was wasted doing some administrative tasks that could be passed to pharmacy technicians to allow the PBP to do more clinical work and ultimately achieve better integration:

*‘… get away from the hospital letters, start the technician role, get more clinics, see more patients that would be for me a better integration.’*

(PBP10)

In addition, GPs revealed that having more full-time PBPs in general practice would allow more activities to be undertaken:

*‘Our practice-based pharmacists are busy … I suppose if we could have more full- time pharmacists that would be fantastic … there’s a huge amount of work that could be done.’*

(GP06)

Practices used different approaches to decide which activities the PBP would undertake. As PBPs were employed by a federation, this could influence what PBPs did. Participants described that there were ‘two bosses’, ‘a blended approach’, or ‘dual function’ to decide the activities of PBPs within general practices (that is, through the key performance indicators set by the federation and the needs of the practice):

*‘Sometimes it can be quite difficult because you nearly have two bosses; you have your federation boss and then you have your practice manager and your lead GP in your doctor surgery.’*

(PBP01)

However, some GPs and PBPs believed that practices should have had more input into how they wanted the PBP to work within the practice:

*‘Full integration for me would really be where the role of the pharmacist is very much driven by the individual practice … so driving on the structure of the practice.’*

(GP05)

Many participants highlighted a lack of awareness of the PBP role by others, including patients as summarised below.

#### GPs’ awareness of the PBP role

GPs and PBPs emphasised that the PBP role had not been clearly defined at the beginning of the initiative. However, they reported this had evolved and was now much clearer:

*‘I think it has become much clearer over time … initially when the practice-based pharmacist was first introduced into the practice, we probably thought* [of] *some of the things that they could do and then after being with us, we were able to see more and more the value of them.’*

(GP08)

Despite this clarity, GPs and PBPs said that the role was not clear to all GPs:

*‘Not to all GPs. I think there are some GPs who … do not let them* [PBPs] *use their skills and their training to their full potential.’*

(GP02)

#### CPs’ awareness of the PBP role

Most PBPs perceived that their role was not entirely clear to CPs; CPs had similar views, but it largely depended on the relationship between the CP and the PBP. CPs’ limited awareness of the role was attributed to the lack of information about a PBP joining a general practice; they only became more aware while working with PBPs on a day-to-day basis:

*‘I think sometimes they* [CPs] *think we don’t do anything … but in a word “no”, I don’t think they know the role.’*

(PBP11)

*‘I’d say it’s not entirely clear … I’m not entirely sure of the role.’*

(CP11)

*‘… we actually built relationships … I think that helped community pharmacists realise what we do.’*

(PBP10)

*‘I don’t think we have been like that for example given any further information regarding what practice pharmacists work where and when … now we can hear what they do from day to day and their roles certainly but probably not initially.’*

(CP04)

#### Patients’ awareness of the PBP role

Most participants believed that patients were largely unaware of the PBP role and did not understand the difference between CPs and PBPs (that is, patients might think that the PBP worked in community pharmacy and did not realise that there was a pharmacist based in the practice):

*‘A lot of patients are not familiar that we have maybe* [a] *pharmacist in the practice … I think a lot of them would not really differentiate between practice pharmacists and community pharmacists.’*

(GP02)

*‘A lot of patients are unaware that practices have practice-based pharmacists, and they think sometimes the practice pharmacist is just someone that works in community pharmacy and provides a prescription …’*

(PBP07)

*‘I would say probably not … you would still have patients that maybe don’t realise that there is a pharmacist based in the surgery …’*

(CP05)

Most GPs and PBPs felt that full integration of PBPs into general practice and promotion of the role would lead to greater awareness so that, in the future, patients could contact the practice to speak to a pharmacist about an acute problem or the practice team could refer patients to the PBP when appropriate rather than to the GP:

*‘So full integration to me would be … they* [patients] *know what we exactly do, what our role is, and that we’re there to help them and they don’t necessarily need to always speak to GP about an issue.’*

(PBP03)

### Theme 2: PBP attributes

Participating GPs and PBPs reported that communication skills were essential for PBPs to undertake their role. Other useful skills related to: consultations, teamwork and leadership, independent working, time management, and information technology functions:

*‘They need good communication skills as they are quite a link between everybody … skills would be team working skills, leadership skills, and independent working skills … consultation skills with dealing with the huge variety of the general public …’*

(GP06)

Furthermore, a number of GPs and PBPs revealed that PBPs would need clinical skills to allow them to undertake their role and would need the confidence to be able to use these skills:

*‘… they need to have basic clinical skills such as being able to take blood pressure …’*

(GP07)

*‘You need like clinical skills to make sure that you’re doing things safely and we need to have the confidence to be able to check hospital medications as well as community medications …’*

(PBP06)

All participating PBPs had previous experience of working in community pharmacy, which was beneficial in understanding how community pharmacy operated and the importance of having good communication skills:

*‘Because I have experience in community pharmacy, I felt confident with what I do now, so I felt confident coming into that role, so certainly having … good communications with both patients and the multidisciplinary team.’*

(PBP08)

However, all PBPs indicated that there were more opportunities to develop and acquire new skills, including practice-based learning compared with when working in a community pharmacy, and, therefore, they were more satisfied with the PBP role:

*‘I think a lot of the people in the post really enjoy it … there are more training opportunities, more development opportunities … a lot of them have moved from community pharmacy and wanted a change … or better work–life balance.’*

(PBP09)

Several GPs and PBPs believed that it would be very beneficial for the PBP to be an independent prescriber. It was noted that the PBPs who were independent prescribers saved GP time because of reduced duplication of work. Moreover, this qualification enhanced PBPs’ confidence and experience:

*‘… that saves us* [GPs] *a lot of time … there was a lot of duplication of work when he* [PBP] *wasn’t able to prescribe …’*

(GP01)

*‘Everyone needs independent prescribing … it’s about building up the level of confidence and experience …’*

(PBP06)

### Theme 3: collaboration and communication

Participants emphasised that teamwork, supported by clear communication and strong relationships, would be key to achieving collaboration. Trust in, and mutual respect for, each HCP’s expertise and individual skills were also reported as factors to facilitate relationship building and, thus, achieving and enhancing collaborative work:

*‘Interprofessional collaboration would mean communication, having a good relationship … developing trust in the people that you’re working with. I do think that’s the way we work here, it’s very much respectful … so that helps enhance team spirit; feeling that everyone is working within a team.’*

(GP06)

Participants reported several benefits of collaboration between GPs, PBPs, and CPs: streamlining healthcare delivery, reduction in duplication of effort, rapid response to each HCP, and improved patient outcomes and safety:

*‘Ultimately collaboration will seek to reduce duplication of effort and ultimately, if we’re working together synergistically for the patient, that will improve patient outcomes … it streamlines everything … and things are done so much quicker …’*

(PBP10)

Most GPs and PBPs identified that having more formal meetings, involving the PBP in practice meetings and social activities, having full-time PBP positions in the general practice, and having designated times to communicate and to meet regularly would enhance communication, working relationships, and consequently achieve better integration:

*‘It* [full integration of PBPs] *is very much focused on regular meetings and to make that protected time for the pharmacist to meet with all members of the team.’*

(GP03)

*‘… as long as pharmacists are involved in practice meetings and decisions and have those relationships, I think there’s not much more that could be done.’*

(PBP09)

Further details on the impact of full- time PBP positions on integration are given below.

#### Development of relationships across the practice team and with patients

Many GPs and PBPs described the importance of having full-time PBP positions in the general practice to develop relationships across the practice team as well as between the PBP and patients:

*‘… each time that a practice pharmacist has come to our practice, we have spent time developing these relationships and then, unfortunately, sometimes they’ve had to move on … and sometimes there’s a reluctance to invest time unless you’re sure that the pharmacist that you’re getting is going to be there for long term.’*

(GP07)

*‘… being permanently employed within a practice is key … it* [PBP role] *needs to be a permanent role … so that GPs get what they want from practice-based pharmacists … and practice-based pharmacists can develop relationships with GPs and patients within the practice as well …’*

(PBP02)

#### Completion of PBPs’ tasks

A number of PBPs highlighted the difficulties of covering too many practices and the effect on some tasks, for example, following up hospital letters:

*‘… you would find it kind of hard to sort of go to one surgery one day and then go to another surgery the next day, we need to be able to build up relationship with other workers in the primary care team, but also be able to build it up with patients, and also some following up things, it just kind of sort of the hospital letters when you are and then that’s you move on to the next surgery …’*

(PBP03)

#### The response to CPs’ queries

Some CPs described occasional delays in response if a PBP was not in the practice on a full-time basis:

*‘… I have noticed that you know a surgery with a practice-based pharmacist, you get* [a] *response much quicker, whereas compared to a surgery that wouldn’t have one or the practice-based pharmacist doesn’t work that day, there will be delay sometimes.’*

(CP04)

PBPs were considered to be in a perfect position to link between different professionals and to act as the first point of contact in general practice for CPs. Many saw PBPs as a ‘central hub–middleman’ between general practice and community pharmacies and between primary and secondary care. CPs felt that it was easier to communicate with the PBP as opposed to GPs who had historically dealt with queries from CPs:

*‘I’m sort of the middleman … I pass all that information into our community pharmacy to make sure that they can order the right items for the patient …’*

(PBP01)

*‘We don’t speak to the GP as much … but they’re* [PBPs] *more the middleman now …’*

(CP10)

*‘Practice-based pharmacists can respond to queries often quicker and more efficient …’*

(CP05)

Participants emphasised the need to have direct access to the PBP (for example, direct telephone line/email) to improve communication:

*‘We don’t have direct access to speak with the practice pharmacist … the best way for them* [practices] *to do this would* [be to] *have a dedicated phone line for the practice-based pharmacist.’*

(CP07)

Participants noted that having shared education, training, and meetings among the multidisciplinary team and involving CPs would be essential to ensure that all HCPs would adhere to the same principles and procedures in patient care. Participants indicated that these activities would contribute to good working relationships, improve collaboration and communication, and increase HCPs’ awareness of the PBP role:

*‘I suppose if we have time to maybe do teaching or learning together, I suppose that would be really helpful … we could all know that we’re all doing the same thing …’*

(GP10)

*‘I think it’s a fantastic idea where you have multidisciplinary groups in the same table … I might bring to the table something that the others don’t, and the others obviously bring something there I don’t see.’*

(CP07)

PBPs emphasised that better collaboration and communication would be achieved by increasing primary care team members’ awareness and patients’ awareness of the PBP role:

*‘I think education is number one there … I think as a first and foremost educating the main people about the role for example GPs. I think that improves the interprofessional sort of collaboration and communication.’*

(PBP01)

### Theme 4: impact on care

GPs and PBPs reported that PBPs reduced GPs’ workload and saved time by conducting activities previously performed by GPs. As a result, this provided the GP with more time to see patients, resulting in better patient care:

*‘They* [PBPs] *have given us more time … we’re not trying to terminate the consultation in order to do the huge pile of paperwork so we can do more work directly with the patient … and that the patient will get the benefit of that.’*

(GP06)

Furthermore, GPs indicated that PBPs were an invaluable source of information, ensuring that GPs were aware of the most current guidelines.

*‘They* [PBPs] *are a great source of information … they are able to sit down and look at the drugs and really take the time to go through them properly … they are very up to date as well on the guidelines, as well quite a lot of time they are keeping us up to date with the guidelines …’*

(GP10)

GPs and PBPs also reported that PBPs’ activities enhanced patient safety as they had more time to focus on hospital discharge letters, chronic disease review clinics, and queries from community pharmacies and secondary care. These activities reduced interruptions experienced by GPs during their work and ultimately reducing the risk of errors. GPs also reported that the PBP was detail oriented:

*‘It has made our life better and safer and more pleasant … it has done that for me when you’re coming into a busy day and they’re at the end of it, you have maybe got sixty letters to process, you know, mistakes are going to be made with tiredness and with just too much volume of stuff … so there’s attention to detail and having a bit more time …’*

(GP03)

Participants indicated that the PBP had a critical role in medicines optimisation, particularly for the older population who were at higher risk of adverse drug events and those with polypharmacy:

*‘I feel like medicines optimisation is kind of our main role … our medication review specifically wants to focus on those patients with polypharmacy … those that are frail and elderly …’*

(PBP06)

## DISCUSSION

### Summary

This qualitative study highlighted that the PBP role had evolved since its introduction across general practice in NI and the PBP role has had a positive impact on GPs, CPs, and patients. Insights were provided into participants’ views on what had contributed to integration (for example, a good GP–PBP collaboration) as well as aspects that required further attention (for example, patients’ awareness of the role) to ensure better and continuing integration of PBPs into general practice.

### Strengths and limitations

The qualitative design and the triad approach provided a more comprehensive overview of the working relationships between the three HCP groups and allowed for an in-depth and thorough understanding of participants’ views. Data were analysed independently by two researchers and decisions were made on the themes through a consensus approach, enhancing the trustworthiness of the findings and in accordance with the COREQ checklist.^16^

A number of limitations should be noted. First, recruitment was limited to one UK geographical region. Most participants were recruited through snowball sampling that may have introduced potential bias. No PBPs were interviewed who had previously worked in hospital pharmacy (that is, all had a community pharmacy experience), which could affect the transferability of the findings. However, despite this, three key HCPs from different NI trust areas were included and their perspectives are broadly similar to those reported in the literature, thereby reinforcing the transferability of these findings.

### Comparison with existing literature

In this study, it was apparent that the PBP role varied between practices and there were several activities undertaken by PBPs that have previously been identified.^12^^,^^24^^,^^26^^,^^31^^–^^40^ However, insufficient time and current workload were perceived as barriers to undertaking some activities and have been noted in other research.^23^^,^^34^^,^^38^^,^^39^ Integrating pharmacy technicians into general practice was suggested as a way to save PBP time to conduct more clinical work and thus make better use of PBP skills. Previous studies have shown that there is the potential to expand the role of pharmacy technicians in the UK to become more involved in the future delivery of medication reviews.^39^^–^^41^

Lack of patient awareness of the role was highlighted by nearly all participants. As the PBP role was relatively new and varied between GP practices, participants believed that patients did not understand the difference between CPs and PBPs, which is consistent with other studies.^21^^,^^25^^,^^42^^–^^44^ Nearly all participants in the current study emphasised the need to properly inform patients and primary care team members about PBPs, including roles and responsibilities, to raise patients’ awareness and therefore encourage the uptake of PBPs’ services, ultimately leading to better integration of the PBP.^20^^,^^34^^,^^38^^,^^39^^,^^42^^,^^44^ Further work to explore patients’ understanding and views of the PBP role in general practice is necessary to corroborate the participants’ concerns in the current study.

Awareness of the PBP role was one of the key aspects that requires further attention to achieve better integration. This current study indicates that, although most GPs were aware of the role, participants reported that a minority were not. Furthermore, the PBP role was not entirely clear to CPs, which has been previously reported.^21^^,^^45^ Clearly defined roles improve collaboration and decrease misunderstandings about responsibilities and authority.^46^^–^^48^ Consistent with the findings in this study, previous studies highlighted that scheduling meetings with individual team members was a common approach to informing the team about the role.^49^^,^^50^

Having full-time PBPs was described as a way to enhance communication. Many highlighted the advantage of having a full-time PBP to build and develop strong relationships and ultimately better integration into a team. This has been reported elsewhere.^23^^,^^24^^,^^33^^,^^34^ Working across many practices could possibly lead to a lack of continuity, thus hindering integration,^11^^,^^40^ while also having an impact on tasks such as managing repeat medication re-authorisation.^39^ A previous study found that patients reported difficulties in arranging appointments with PBPs who covered multiple practices.^23^^,^^42^

The findings of this current study have indicated that PBPs are ideally placed to use their skills and knowledge to help the GP practices and liaise with CPs. Evidence has shown that PBPs provide valuable services to ease the burden on the GP and reduce patient waiting times.^51^^–^^53^ In the current study, it was perceived that PBPs had an impact not only on GP workload but also on patient safety and care, and medicines optimisation. These are reassuring findings as, for example, 20% of hospital admissions among older people are the result of adverse effects of prescribed medications.^54^

### Implications for practice

There are a number of recommendations for practices based on the results of this study. As the role of the PBP is expected to expand to include more clinical patient-facing roles, pharmacists must be prepared for this. New standards for the initial education and training of pharmacists across the UK have been recently introduced, for example, qualified to undertake independent prescribing from the point of registration.^55^ Furthermore, this study revealed that PBPs would need clinical skills to allow them to undertake their role and would need the confidence to be able to use these skills. A recently published Delphi study has produced a core set of clinical skills required for pharmacist prescribers working in general practice that could inform training.^56^ However, expansion of the role and increasing patient awareness of the role may exacerbate workload issues. Therefore, having more PBPs and pharmacy technicians in general practice may help to address these demands.

A number of policies have been launched in the UK to expand the current integration of PBPs in general practice.^57^^–^^59^ Therefore, the findings from this study provide valuable insights for policymakers, practice managers, and service commissioners into what is required to ensure better, efficient, and smooth integration of PBPs. The findings from this study may be useful in countries where consideration is being given to the development of pharmacist services in general practice.
